# The interplay between pathogens and Atg8 family proteins: thousand‐faced interactions

**DOI:** 10.1002/2211-5463.13318

**Published:** 2021-11-17

**Authors:** Dávid Tóth, Gábor V. Horváth, Gábor Juhász

**Affiliations:** ^1^ Institute of Genetics Biological Research Centre Szeged Hungary; ^2^ Department of Anatomy, Cell and Developmental Biology Eötvös Loránd University Budapest Hungary

**Keywords:** GABARAP, hfAIM, iLIR, LC3, LIR motif, SARS‐CoV‐2

## Abstract

Autophagy is an intracellular degradation and recycling process that can also remove pathogenic intracellular bacteria and viruses from within cells (referred to as xenophagy) and activate the adaptive immune responses. But autophagy—especially Atg proteins including Atg8 family members—can also have proviral and probacterial effects. In this review, we summarize known interactions of bacterial, parasitic, and viral proteins with Atg8 family proteins and the outcome of these interactions on pathogen replication, autophagy, or mitophagy. We discuss the value of prediction software and the research methodology in the study of pathogen protein‐Atg8 family protein interactions, with selected examples of potential LC3‐interacting region motif‐containing SARS‐CoV‐2 proteins.

## Graphical abstract

A possible workflow for the interaction analysis of pathogen proteins and Atg8 family proteins. In this review, we discuss recent advances in the field, the value of prediction software and the research methodology in the study of pathogen‐Atg8 family protein interactions, with selected examples of potential LC3‐interacting region motif containing SARS‐CoV‐2 proteins.

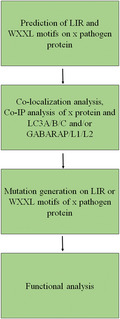

**Abbreviations**
DMV: double‐membrane vesicle
EDEM1: ER degradation‐enhancing alpha‐mannosidase‐like protein 1
ERAD: endoplasmic reticulum‐associated protein degradation
GABARAP: gamma‐aminobutyric acid receptor‐associated protein
hfAIM: high‐fidelity autophagy‐associated atg8‐interacting motifs detecting tool
LC3: microtubule‐associated protein 1A/1B‐light‐chain 3
LIR: LC3‐interacting region
mAtg8: mammalian autophagy‐related protein 8
NSP: nonstructural protein
PSSM: position‐specific scoring matrix
PV: parasitophorous vacuole
SEL1L: protein sel‐1 homolog 1

During the process of macroautophagy (hereafter referred to as autophagy), the various substrates in the cytosol are more or less randomly entrapped in double‐membrane‐bound vesicles called autophagosomes, and their contents are degraded after fusion with lysosomes. This process is required for proper removal of protein aggregates and damaged organelles and involves lysosomal hydrolases that degrade proteins, lipids, nucleic acids, and carbohydrates. The resulting breakdown products are then transported back to the cytosol and contribute to the replenishment of the cellular metabolic pool [1]. Selective autophagy is required for specifically removing components such as protein aggregates and damaged or unneeded organelles. Different types of selective autophagy can be distinguished according to the cargo: mitophagy (mitochondria), pexophagy (peroxisomes), ribophagy (ribosomes), reticulophagy (endoplasmic reticulum), and nucleophagy (parts of the nucleus). Interestingly, there is a type of selective autophagy called xenophagy, which precisely recognizes intracellular microorganisms and targets them to autophagosomes for degradation [2]. Evolutionarily conserved autophagy‐related proteins (Atgs) that were mostly discovered in yeast play a key role in the main pathway of autophagy. Of these, small Atg8 family proteins [including microtubule‐associated protein 1A/1B‐light‐chain 3 (LC3) and gamma‐aminobutyric acid receptor‐associated protein (GABARAP) subfamilies in mammals, collectively referred to as mammalian autophagy‐related protein 8 (mAtg8s)] should be highlighted, which in their lipid‐conjugated form are localized on the surface of pre‐autophagosomal membranes (phagophores) and mature autophagosomes [3]. As our review focuses on mAtg8 interactions with pathogen proteins, it is worth emphasizing that seven human orthologs of yeast Atg8 exist (LC3A, LC3B, LC3B2, LC3C, GABARAP, GABARAPL1, and GABARAPL2) [4].

The interaction of a certain protein with mAtg8 proteins require a short linear motif (LIR), the LC3‐interacting region [5]. The most accurate definition of the LC3‐interacting region (LIR) motif was shaped by Wirth et al. [6]: LIR is a specific region of a protein, which is recognized by ATG8 proteins. The canonical LIR motif is a small Θ0‐X1‐X2‐Γ3 motif, where Θ represents an aromatic residue (W/F/Y) and Γ an aliphatic residue (L/V/I) and X represents any amino acid (aa) [6]. In our review, we highlight well‐characterized pathogen protein interactions with mAtg8 proteins (summarized in Table 1). Our examples show the utility of predictive analysis software, the wide variety of methods to study these interactions and the impact of research on mAtg8 binding to pathogen proteins. We also demonstrate some of our predictions and results on the LIR motif‐containing SARS‐CoV‐2 proteins to show the effectiveness of *in silico* methods developed to predict the LIR motifs of cellular proteins for the study of viral protein‐mAtg8 binding.

**Table 1:** Summary of the known pathogen protein interactions with mAtg8 family members.

Type of pathogen	Pathogen name	Pathogen protein name	LIR motif	Interacting Atg8 family member	General outcome of the pathogen protein ‐ Atg8 interaction	Outcome on autophagy/mitophagy	Ref
Bacterium	*Legionella pneumophila*	RavZ	LIR2: residues 27–32	LC3	Interference with the lipidation of mAtg8 proteins Cleavage of lipidated mAtg8 (mAtg8‐PE) from the autophagosome membrane.	Autophagy inhibition	[19, 20, 21, 22, 23, 24]
	*Burkholderia pseudomallei*	BPSL2203	Potential LIR motifs: aa. 315–320, aa. 380–385	LC3	Not observed	Not observed	[31]
	*Salmonella enterica* serovar Typhimurium SL1344	YhjJ	(464)ATWDEI(471)	LC3B	Facilitate autophagic degradation of Salmonella.	Autophagy induction/inhibition	[32]
	*Listeria monocytogenes*	Listeriolysin O	Not identified	Indirect	Targets NLRX1, and its LIR motif.	Mitophagy activation	[33]
Eukaryotic pathogen	*Plasmodium berghei*	UIS3	Lacks a canonical LIR	LC3	Blocking autophagy proteins from associating with LC3	Autophagy inhibition	[35]
Virus	Influenza A virus	Matrix 2	(90)FVSI(95)	LC3	LC3 relocalization to the plasma membrane Filamentous budding and virus stability	Autophagy induction	[40]
	EBV	BALF1	(145)WSRL(150)	LC3	Autophagy induction	Autophagy induction	[41]
	Human immunodeficiency virus	Vif	Independent	LC3A, LC3B, LC3C, GABARAP, GABARAPL1, GABARAPL2	Restrict innate antiviral mechanisms via inhibiting autophagy	Autophagy inhibition	[44]
		Nef	Independent, critical amino acids: S53 and F62	GABARAP, GABARAPL1, GABARAPL2	Required for Nef plasma membrane localization	Not observed	[58]
	Human parainfluenza virus type 3	Matrix	Independent; critical amino acid: K295	LC3	Activates mitophagy to inhibit the type 1 interferon response.	Mitophagy activation	[46]
	Mouse hepatitis virus	nsp2/nsp3	Not identified	LC3‐I	EDEMosome/DMV formation, propagate replication	Autophagy independent mechanism	[51]
	Porcine reproductive and respiratory syndrome virus	NSP2	Not identified	LC3	Promote replication complex formation	Autophagy activation	[52]
	Japanese encephalitis virus	NS1	Not identified	Indirect interaction with LC3	EDEMosome/DMV formation, propagate replication	Autophagy activation	[54]
	Borna disease virus	Phosphoprotein	Critical amino acids: aa. 174–201	GABARAP	Delays the trafficking of GABAA receptor to the cell membrane Blocks GABA‐induced currents	Not observed	[56]

It is worth mentioning that selective autophagy pathways including xenophagy usually also rely on the interaction of cargo receptors with mAtg8 family proteins, reviewed in detail elsewhere [7, 8, 9].

## Bacterial proteins: the impact of their interaction with mAtg8s

### *Legionella pneumophila* RavZ protein: the best studied bacterial tool for manipulating mAtg8 protein functions

*Legionella pneumophila* is a Gram‐negative bacterium of the order Legionellales that causes Legionnaires' disease (Legionellosis). Other bacteria in this order are also pathogens, although the symptoms they cause (Pontiac fever) are much milder than those of Legionellosis infection, which often involves fatal pneumonia [10]. *Legionella* *pneumophila* secretes more than 300 identified effector proteins into the infected cell through its Dot/Icm secretory system, supporting the survival and multiplication of the bacterium in the cell [11]. *Legionella* can transform the membrane transport processes and the endomembrane system of the host cell in order to create special vacuoles that correspond to its own replication [12]. Understanding the mechanism by which *L*. *pneumophila* influences autophagy started with the fundamental work of Choy et al. [13]: They demonstrated that HEK293 cells infected with the virulent Philadelphia‐1 strain had significantly lower levels of lipidated LC3 (LC3‐II) compared with uninfected cells. When an isogenic *Legionella dotA* mutant (which does not have a functional Dot / Icm secretion system) was used for infection, the amount of LC3‐II was found to be the same as that of uninfected cells. Examination of isogenic *Legionella* strains carrying large chromosomal deletions revealed that the RavZ effector protein was able to inhibit the autophagic process. To elucidate the mechanism involved, the effect of purified RavZ protein was studied in an *in vitro* system using synthetic liposomes containing recombinant PE‐conjugated human Atg8 family protein (GABARAP‐L1). The presence of RavZ protein caused complete de‐lipidation of the lipid‐conjugated form, demonstrating that inhibition of autophagy is a direct result of interference with the lipidation of mAtg8 proteins.

The importance of this work also lies in the fact that although the formation of autophagosomes was observed in cells infected with the *∆ravZ mutant Legionella*, LC3‐positive membranes were not formed around vacuoles containing the RavZ‐deficient *Legionella* mutant. This suggested that there are other Legionella effectors that prevent bacterial‐containing vacuoles from being degraded by autophagy. This theory was later supported by a study of the Lpg1137 effector of *Legionella*. This effector is a serine protease that targets mitochondria and the ER membrane associated with them. Lpg1137 degrades the syntaxin 17 SNARE protein, an essential component of the late endosome/lysosome‐autophagosome fusion machinery [14, 15]. The protease action of Lpg1137 inhibits not only autophagy but also staurosporine‐induced apoptosis [16, 17]. A recent study suggests an even more complex mechanism. Co‐infection experiments of Omotade and Roy [17] with *Listeria monocytogenes* and *L*. *pneumophila* showed that autophagy of *L*. *monocytogenes*‐containing vacuoles was inhibited in a RavZ‐dependent manner by *L*. *pneumophila* infection, whereas with a strain of *L*. *pneumophila* in which all SidE family effector genes were deleted, the bacterium‐containing vacuoles are still not targets for the mechanism of host cell xenophagy. They suggest that *L*. *pneumophila* has at least two systems to prevent the breakdown of bacteria‐containing vacuoles. One is the RavZ‐dependent pathway, which generally inhibits autophagy in the cell, and the second one is a cis‐acting mechanism that prevents autophagy adapters from binding to the ubiquitin‐labeled surface of *L*. *pneumophila*‐containing vacuoles [17].

### RavZ structure–function studies

Soon after the discovery of RavZ, structural studies have begun to explore the mechanism of its action. Horenkamp et al. [18] showed that the catalytic domain of the protein is structurally similar to the Ulp family of deubiquitinating enzymes and it has a PI3P‐binding region at its C terminus; both playing a role in autophagic membrane binding [18]. Bond formation depends on the degree of membrane curvature, preferentially binding to the highly bent domains of the autophagosomal intermediate membranes.

The catalytic activity of RavZ involves the cleavage of lipidated mAtg8 (mAtg8‐PE) from the autophagosome membrane. Amino acid sequence analysis of the protein suggested the presence of two LIR motifs at the N terminus (LIR1: residues 14–19 and LIR2: residues 27–32), while one LIR motif was present at the C terminus of the protein (residues 433–438) [19]. The two LIR motifs located in tandem at the N terminus have a noncanonical β‐sheet conformation, and although only the LIR2 motif is likely to be involved in LC3 binding, this conformation may allow for stronger association with other mAtg8 proteins [19]. Based on the three‐dimensional model, due to steric hindrance, the N‐terminal tandem LIR motif is only able to bind one LC3 molecule [19]. In addition to these motifs, the catalytic (CAT) domain is found in RavZ, which is structurally similar to Ulp proteases and contains the catalytic triad His176‐Asp197‐Cys258 required for activity. [18, 20] The MT domain responsible for PI3P binding, a phospholipid that is present in high concentrations in autophagosomal membranes, is localized at the C terminus of RavZ [19]. Summarizing the structural information, the following model of the deconjugation activity of the RavZ protein is drawn: N‐ and C‐terminal LIR motifs are associated with LC3‐PE molecules, and the CAT domain delipidates the LC3‐PE molecule on the membrane. This is described by Kwon et al. and they proposed a ‘Tethering and Cut’ model, which also seems plausible based on SAXS (small‐angle X‐ray scattering) data [20]. Almost at the same time as this model, another explanation, ‘Lift and Cut’, was born. In this model, the task of performing deconjugation is shared between the lipid and protein binding motifs of RavZ, and the CAT domain is responsible for catalytic activity. The lipid extraction function of RavZ (predictable based on structural similarity to the yeast Sec14 phospholipid transfer proteins) is responsible for the extraction of the LC3‐PE conjugate from the autophagosome membrane, while the cysteine protease activity of the CAT domain performs cleavage. In this model, only the N‐terminal LIR2 motif plays an important role in LC3 binding [21, 22].

Both models require further investigation: The first does not consider the membrane‐binding property of RavZ, and the second does not explain why deletions or mutations in any of the LIR motifs lead to a decrease in RavZ activity [20, 21]. The results of a recent study suggest a conciliatory but complex solution in which the RavZ protein has different strategies for deactivating the early and late stages of autophagy [23]. The accumulation of PI3P in early autophagosome membranes is characteristic, while lipidated mAtg8 proteins are constitutive autophagosomes markers: They are present until lysosomal degradation. The RavZ protein uses this duality to generally inhibit the formation of autophagic membranes. Thus, one of the bases of the mechanism is the recognition of PI3P‐rich membranes, which is also mediated in RavZ LIR mutants through the MT domain; the other cornerstone is the recognition of mAtg8 proteins by LIR motifs, which the RavZ(ΔMT) mutant is capable of. Both processes lead to decreased lipidation (however, with obviously different efficiencies), resulting in inhibition of autophagy at different stages. A detailed study of the mechanism of action of RavZ would not have been possible without the elaboration of the synthesis of lipidated LC3 proteins used in the experiments. Yang et al. describe a semi‐synthetic method for the preparation of various C‐terminally modified LC3 proteins by the expressed protein ligation (EPL) technique followed by direct aminolysis of protein thioesters [24]. In this way, a broad spectrum of C‐terminally lipidated and yet soluble LC3 proteins was generated.

The research detailed above has had a significant impact on the discovery of the function of LIR motifs in certain cellular Atg proteins. A good example of this is the study of Kauffmann et al. comparing the deconjugation activity of RavZ and the function of the four human Atg4 proteins with similar activity [25]. RavZ protease was found to be active only on membrane‐bound, lipidated mAtg8 proteins, whereas Atg4 proteins can cleave both unconjugated and lipid‐conjugated substrates in line with their cellular function. The activity on soluble LC3 and GABARAP proteins is primarily due to Atg4B, while all four Atg4 proteins are involved in the cleavage of membrane‐bound, lipidated LC3 with nearly similar activity. Mutation of the N‐terminal LIR motif does not result in a significant decrease in the recognition of either soluble or membrane‐bound substrates, whereas mutation of the C‐terminal LIR motif primarily reduces membrane‐bound LC3 processing. Rasmussen et al. similarly argue for the role of the C‐terminal LIR in the Atg4B‐GABARAPL1 interaction based on crystal structure data. An unexpected role for this LIR motif is described in this work: It stabilizes soluble (nonlipidated) GABARAP and GABARAPL1 proteins in cells [26].

The results of the detailed study of RavZ also allowed for technological development. Park et al. [27] demonstrated how a catalytically inactive mutant of RavZ (replacing the CAT domain with GFP) can be used to generate a marker that well marks autophagosomal membranes. This work demonstrated that membrane binding is indeed a combined effect of LIR motifs and the PI3P‐binding MT domain, and also that this combination is more efficient than the LIR‐based sensors used previously. To distinguish between the LC3 and GABARAP subfamilies, specific probes were created by replacing the LIR motifs of the RavZ protein with those of Fyco1 or ULK2, respectively.

### Other bacterial proteins with functional LIR motifs

Because the subject of the present review is the interaction of LIR motif‐containing pathogen proteins with LC3/GABARAP proteins and their effect on the autophagic processes, we do not discuss the many published results examining LC3 association with pathogen‐containing vacuoles. In the first example, we can only assume the role of the LIR motif(s) in the bacterial protein‐LC3 interaction, but we do so with a good reason.

The Gram‐negative pathogen *Burkholderia pseudomallei*, the causative agent of melioidosis common in South‐East Asia and Northern Australia, uses a number of virulence factors in the infection of mammalian cells. One such factor (similarly to *Legionella*) is the type three secretory system (TTSS), which secretes and transports bacterial effectors to the host cell. D'Cruze et al. [28] examined a *bpscN* mutant of *B*. *pseudomallei*, a gene encoding the ATPase protein of the TTSS1 system. This mutant showed significantly reduced virulence in a mouse model of respiratory melioidosis, as well as reduced viability and replication in murine macrophage‐like RAW264.7 cells. These phenomena were associated with increased colocalization of *bpscN* mutant bacteria with the GFP‐LC3 autophagosomal marker, and it is known that induction of autophagy and LC3‐associated phagocytosis play an important role in the control of *B*. *pseudomallei* multiplication within the cell [29, 30]. Examination of the *Burkholderia* protein‐LC3 interaction by the liquid chromatography tandem‐mass spectrometry (LC–MS/MS) method resulted in the identification of a new interaction partner (Joompa et al. [31]): the BPSL2203 protein, which is a bacterial ABC transporter periplasmic substrate‐binding protein [30]. Although no further analysis of this protein has been performed yet, according to our own analysis, it also contains two LIR motifs (aa. 315–320, aa. 380–385) that are recognized by both iLIR and high‐fidelity autophagy‐associated atg8‐interacting motifs detecting tool (hfAIM) prediction software. With the knowledge that the BPSL2203 protein indeed exhibits LC3 association, analyzing the role of these LIR motifs requires further but relatively straightforward experimental work.

The *in silico* analyses mentioned above can help in the understanding of how different pathogens may modify autophagic processes for their own purposes. Such a large‐scale analysis was presented by Sudhakar et al. [32] who predicted the binding motifs of selective autophagy receptors (SQSTM1/p62, CALCOCO2/NDP52) and MAP1LC3/LC3 in effector proteins of 56 pathogenic bacterial species. It is worth mentioning for the purposes of the present study that an example supported by experimental data was also presented: the YhjJ zinc protease of *Salmonella enterica* serovar Typhimurium SL1344, which is expressed upon infection of the host cell. Based on the prediction, the protein contains an LC3B‐interacting LIR motif, and GST‐LC3B and 6xHis‐YhjJ pull‐down experiments demonstrated the existence of this interaction. The observed decrease in the number of LC3B‐positive structures in the cell and the increase in the number of p62‐positive *Salmonella*‐containing vacuoles in the *Salmonella ΔyhjJ* strain showed that this secreted protease is indeed able to influence host cell autophagic processes.

The strategy used by *L. monocytogenes* to induce mitophagy draws attention to the fact that the autophagy‐modifying effect of pathogens may also depend indirectly on the LIR motif‐containing protein‐LC3 interaction. Zhang et al. [33] described for the first time the mitophagy‐inducing effect of a bacterial pathogen, showing that mitophagy induced by *L*. *monocytogenes* has a survival advantage for the bacterium. The secreted Listeriolysin O (LLO, a pore‐forming hemolysin produced by the bacterium), is considered a virulence factor, since it is crucial for the propagation of *L*. *monocytogenes* [34]. LLO targets the recently identified mitophagy receptor NLRX1, and its LIR motif (due to its interaction with LC3) induces mitophagy. This process reduces the amount of ROS produced by mitochondria, favoring the cellular environment for bacterial growth.

### Beyond bacterial pathogens: a promising example from the world of intracellular parasites

How important is the LC3/GABARAP interaction mediated by the LIR motifs of pathogen proteins for drug discovery? To answer this question, we now need to go beyond viral/bacterial pathogens and examine an example from intracellular parasites.

*Plasmodium*, the causative agent of malaria, replicates mainly within the host liver cells in a membrane‐bound, parasitophorous vacuole (PV); this protects the intracellular parasite from the degradative mechanisms. The main mechanism that inhibits parasite proliferation is xenophagy, which directs LC3‐coated PVs toward lysosomal degradation. Real et al. showed that the survival of *Plasmodium berghei* parasites infecting liver cells depends on the UIS3 transmembrane protein, which interacts with the host cell LC3 protein [35]. This strong interaction prevents other autophagy proteins from associating with LC3, so UIS3 acts as a general autophagy inhibitor. Already in this work, the authors suggested the blocking of the UIS3‐LC3 interaction as a direction for the development of antimalarial drugs [35, 36].

Finally, a successful strategy was described by Setua et al. [37]. The three‐dimensional structure of the UIS3‐LC3 complex was required first for drug efficacy studies. With this knowledge, an *in silico* small molecule binding analysis (virtual library screen, VLS) could be performed involving more than 23 million compounds. This led to the selection of 21 candidate molecules that could bind to the UIS3‐LC3 complex based on prediction. For the subsequent phenotypic screen (PHS), a recombinant *P*. *berghei* strain was used in which the *UIS3* gene was replaced with the corresponding gene of *P. falciparum*. The phenotypic screen in Huh7 cells confirmed that the previously preferred compound C4 (4 ‐ {[4‐ (4‐ {5‐ [3‐(trifluoromethyl) phenyl] −1,2,4‐oxadiazol‐3‐yl} benzyl) piperazino] carbonyl} benzonitrile)) was the most effective in reducing parasite number, and the binding of C4 to the UIS3‐LC3 complex has been demonstrated by Isothermal Calorimetry (ITC). The study also demonstrated that the antiparasitic effect of C4 requires a host cell autophagic mechanism, and the application of C4 at an effective concentration did not alter the normal autophagic processes of the cell.

This is the best demonstration that accurate mapping of the LIR motifs of pathogenic proteins, the detailed analysis of their interaction with mAtg8 proteins, and finally *in silico* docking analysis of small molecules can also be an effective strategy for the development of new antibacterial/antiviral compounds.

## How and why viral proteins interact with mAtg8 proteins?

The aim of this section is to clarify how various viral strategies have been developed to modulate and use mAtg8s. Four groups were separated based on the viral protein and mAtg8 interactions:

1. LC3‐interacting region motif‐dependent use of LC3 among viruses.
2. LC3‐interacting region‐independent use of LC3.
3. Double‐membrane vesicle (DMV) formation via viral proteins and LC3 interaction: the best studied interplay.
4. GABARAPs, which are crucial interaction partners of viral proteins.

### LC3‐interacting region motif‐dependent use of LC3 among viruses

Although powerful LIR motif prediction software programs are available and the methods to characterize protein–protein interactions are well described, only two viral proteins have been reported with practically relevant LIR‐dependent LC3 interactions. One of them is influenza A virus matrix 2 (IAV M2) protein, and the other is Epstein–Barr virus (EBV) BALF1 protein.

The first confirmed connection of IAV to autophagy was the finding that Matrix 2 (M2) ion‐channel protein blocks the fusion of autophagosomes and lysosomes [38]. Surprisingly, fluorescent microscopy and immunogold EM experiments showed that plasma membrane‐redirected LC3 formed diffuse cytoplasmic signals in IAV‐infected cells. Furthermore, infection with a virus deficient in M2 did not relocalize LC3 to the plasma membrane, which indicates that influenza A virus caused the plasma membrane localization of LC3 via its Matrix 2 (M2) protein. In IAV M2 protein, an FVSI (WxxL) motif‐containing ß strand was predicted that matched the consensus LIR motif [39]. Using the LUMIER binding assay, it was shown that wild‐type M2 protein bound to LC3, whereas M2 without LIR motif (M2ΔLIR) and LIR mutants (M2 F91S, M2 V92S, and M2 I94S) failed to bind LC3. Furthermore, LC3 relocalization to the plasma membrane took place actively in cells infected with viruses with functional M2 LIR motifs, but not in those infected with viruses without M2 LIR motif (M2ΔLIR) or with viruses encoding mutated LIR motifs unable to bind LC3 (M2 F91S and M2 I94S).

What might be the biological impact of the M2 and LC3 interaction? It was precisely elucidated that the LIR motif of IAV M2 protein is required for not only the normal filamentous budding of IAV, but also for the stability of Influenza A virions [40].

Epstein–Barr virus encodes BALF0/1, a viral Bcl‐2 homolog (vBcl‐2) protein. HA‐tagged BALF1 formed cytoplasmic dots that colocalized with GFP‐LC3‐positive vesicles and with endogenous LC3 containing vesicles in HeLa cells. Sequence analysis of BALF1 for [W/F/Y]xx[L/I/V] LIR consensus sequence found a potential LIR motif between amino acids 146 to 149 (146‐WSRL‐149) [41]. To understand the importance of this in the interaction of BALF1 and LC3, single (W146A) and double (W146A and L149A) BALF1 LIR mutants were generated with site‐directed mutagenesis. Both mutations dramatically changed the subcellular localization of BALF1 and produced partial (W146A) or total (W146A and L149A) relocalization of the modified proteins into the nucleus. It was reported that both mutations (especially the double mutant) strongly reduced BALF1's ability to stimulate autophagosome formation as indicated by the low average number of autophagosomes (GFP‐LC3 puncta). As their main conclusion, the authors suggested that the LIR motif of BALF1 mediates its targeting to GFP‐LC3 vesicles and its autophagy‐inducing ability [41].

### LC3‐interacting region‐independent use of LC3

Here, we summarize those viral proteins which directly or indirectly interact with mAtg8s independently of the presence of the LIR motif. As prediction and screening of this type of interaction is challenging, there may be additional viral proteins interacting with mAtg8 in a LIR‐independent manner.

One of the accessory proteins of HIV‐1 is the viral infectivity factor (Vif). Vif binds to the APOBEC3G (A3G) antiviral enzyme and promotes the poly‐ubiquitination and proteasomal degradation of A3G, which restricts the incorporation of A3G in the newly formed virions [42].

GST‐pull‐down experiments revealed binding of Vif to all Atg8 family members (LC3s and GABARAPs) *in vitro*. The interactions with LC3A, LC3B, and LC3C were confirmed *in vivo*.

Three putative LIR motifs (LIR1: WKRL; LIR2: YWGL; LIR3: YLAL) were predicted as potentially responsible for the interaction. By changing the first amino acids to alanine (LIR1: W→A; LIR2: Y→A; LIR3: Y→A), single, double, and triple mutants were generated and tested, as first introduced by Pankiv et al. [43], who showed that the replacement of the first amino acid of each putative LIR sequence by alanine results in a type of LIR mutation that abolishes the interaction with LC3. Surprisingly, the LIR motifs were not involved in the interaction with LC3. After more precise prediction and generation of deletion mutations in the C‐terminal part of Vif, the authors suggested the region between 144 and 159 amino acids inside the SOCS box‐like motif as the region responsible for this interaction.

Reverse experiments were also performed, as the authors were interested in both the responsible region and the form of LC3 in the context of the Vif and mAtg8 interaction. GST‐pull‐down experiments indicated that viral infectivity factor binds to pro‐LC3B independently of the FxL motif of pro‐LC3B. Atg4‐cleaved LC3B and glycine 120 of LC3B are crucial for the interaction. Importantly, the binding of Vif to LC3‐I is stronger than to LC3‐II, indicating Vif's preference for LC3‐I. This may explain autophagy inhibition during HIV‐1 infection in earlier studies [44].

Human parainfluenza virus type 3 (HPIV3) matrix (M) protein activates mitophagy to block the type 1 interferon response through interactions with TUFM (mitochondrial Tu translation elongation factor) and LC3. The interaction with TUFM is required to translocate HPIV3 to the mitochondria, while the connection with LC3 is responsible for mitophagosome formation [46].

M protein colocalization with GFP‐LC3 was first shown by Zhang et al. [45]. CoIP and GST‐pull‐down assays confirmed the physical interaction of M protein with HA‐LC3, GFP‐LC3, and endogenous LC3 in HEK293T cells.

While the authors did not find a classical LIR (W/YxxL/I) motif in M protein, a series of M deletion mutants were tested by CoIP to elucidate which region of M protein is critical for the interaction with LC3. As a result, the deletion of C‐terminal 41–80 residues of M (MΔC41–80) strongly disrupted M interaction with LC3 and failed to induce mitophagy, which means that the interaction of M with LC3 is also essential for M protein‐induced mitophagy. Furthermore, the authors identified a critical amino acid in the M protein: K295. The M K295A mutant neither binds to LC3 nor activates mitophagy.

Zhang et al. showed that M protein is ubiquitinated. The ubiquitination of HPIV3 M protein is essential for M protein‐mediated VLP (virus‐like particle) production [47]. This raises the question of whether the ubiquitination of M protein is essential for its interaction with LC3. It was clearly demonstrated that ubiquitination of the M K295A mutant was reduced compared to wild‐type M. Moreover, in cells treated with MG132 (a drug that decreases free ubiquitin level via inhibition of the proteasome), the interaction of M protein with LC3 significantly decreased but it did not influence the interaction of M with TUFM [46].

### Double‐membrane vesicle formation via viral proteins and LC3 interaction: the best studied interplay

Positive‐strand RNA viruses exploit and rearrange cellular membranes to create their own replicative organelles. These organelles form a protective platform for viral RNA replication and transcription [48]. It is worth mentioning that SARS‐CoV‐2 performs a similar strategy for replication and transcription [49]. Noack et al. [50] suggested a detailed model on how these viruses exploit the endoplasmic reticulum‐associated protein degradation (ERAD) tuning pathway to mediate DMV formation.

Mouse hepatitis virus (MHV) infection causes—independently of autophagy—the accumulation of ER degradation‐enhancing alpha‐mannosidase‐like protein 1 (EDEM1), osteosarcoma amplified 9, and LC3‐I in the DMVs, as demonstrated by confocal microscopy, electron microscopy, and continuous Optiprep gradient analyses. The siRNA‐mediated silencing of LC3A and LC3B protected the cells from MHV infection, and in the absence of LC3, the nucleocapsid (N) protein level and TCID50 (Median Tissue Culture Infectious Dose) value significantly reduced and DMVs did not form. In conclusion, MHV hijacks LC3‐I and EDEM1‐positive EDEMosomes and ERAD regulators to propagate its own replication [51].

Two nonstructural proteins (NSP2 and NSP3) of porcine reproductive and respiratory syndrome virus (PRRSV) form viral replication complexes. Confocal microscopy analysis suggested the colocalization of NSP2 and LC3 in MARC‐145 cells. Continuous density gradient centrifugation confirmed the association of PRRSV NSP2 with LC3 and the ER marker PDI, suggesting that PRRSV RNA replication may happen on the membranes of autophagosome‐like vesicles. Thus, the autophagy machinery can promote PRRSV replication complex formation [52]. The authors did not study the possible involvement of the ERAD tuning pathway and mAtg8s in the process.

The dsRNA of equine arteritis virus (EAV) also showed colocalization with LC3‐I and EDEM1 during the entire course of the infection, independently of the presence of Atg7, the E1‐like enzyme required for LC3 lipidation. However, when only EAV NSP2‐3 proteins were expressed, membrane rearrangements appeared that did not colocalize with LC3 and EDEM1. RNA interference‐mediated reduction of LC3A and LC3B levels decreased replication of EAV, based on reduced nucleocapsid (N) protein level, lower TCID50 value, and, most importantly, the absence of dsRNA that marks DMVs. Moreover, the ectopic expression of nonlipidated LC3 restored EAV replication in LC3‐silenced cells. Thus, the authors concluded that the nonlipidated form of LC3 (LC3‐I) is required for effective replication of EAV [53].

Japanese encephalitis virus (JEV)‐infected cells showed a remarkable overlap between viral NS1 (nonstructural‐1) and endogenous LC3‐I (nonlipidated LC3) independently of functional autophagy, but not with GFP‐LC3 (autophagosomes) during the entire infection. To exclude the connection of NS1 with autophagy, convincing colocalization analyses were performed with LAMP1 and Lysotracker Red staining, and these showed no overlap. Of note, when HA‐tagged NS1 was expressed alone it did not colocalize with LC3‐positive structures, suggesting indirect interaction of the viral protein and mAtg8. Furthermore, it was precisely shown by microscopy and Optiprep gradient centrifugation methods that JEV NS1 associates with EDEM1. These data indicate that virus replication takes place on EDEM1‐ and LC3‐I‐positive EDEMosomes. Moreover, the deprivation of ERAD regulators, like EDEM1 and Protein sel‐1 homolog 1 (SEL1L), diminished JEV replication. LC3 silencing also inhibited JEV replication, as JEV RNA levels and virus titers significantly reduced [54].

Coxsackievirus B3 (CVB3) host membrane modification strategy is a good example of viral strategies. To test how CVB3 modifies host membranes to form replication platforms, Alirezaei et al. generated three recombinant viruses (proLC3‐CVB3, proLC3^G120A^‐CVB3, and ATG4B^C74A^‐CVB3). All three virus‐encoded autophagy‐related proteins enhanced viral replication and caused severe pancreatitis. In the case of proLC3‐CVB3 recombinant virus, it was confirmed that viral LC3 increased the autophagy‐dependent formation of LC3‐II coated DMVs. The proLC3^G120A^‐CVB3 bound to SEL1L thus exploited the ERAD pathway to enhance autophagy‐independent LC3‐coated DMV development. ATG4B^C74A^‐CVB3 virus did not produce LC3 due to a block of LC3 processing by the dominant‐negative Atg4 protease mutant, and in the absence of lipidated LC3, phagophore maturation was disrupted and replication scaffolds were produced independently of autophagy. Based on these findings, the authors suggested that CVB3 can hijack host membranes from ER in three independent ways [55]. It is worth emphasizing that the application of recombinant viruses provided precise information about Coxsackievirus B3 membrane hijacking strategies. Another interesting point of this study was that the virus can exploit different membrane sources, which means that the drug‐mediated inhibition of a single pathway would not necessarily inhibit viral replication.

The authors emphasize the importance of LIR motif‐based interaction analyses because it is unclear whether NSPs directly interact with LC3 through a LIR motif or not. The use of LIR mutants could also explain the direct regulatory role of LC3 in DMV formation. It is also worth considering the analysis of the potential involvement of GABARAPs in the process.

### GABARAPs are crucial interaction partners of viral proteins

In this section, those viral proteins that interact with GABARAP proteins potentially through a GABARAP‐preferring LIR motif, also known as GABARAP interacting region (GIR), are highlighted.

Borna disease virus (BDV) phosphoprotein (P) is vital for virus replication as a viral RNA‐dependent RNA polymerase cofactor. Direct binding between GABARAP and P protein was shown by CoIP, His pull‐down, and mammalian two‐hybrid assays. Both in BDV‐infected cells and P protein‐transfected cells, GABARAP colocalized with P protein and this interaction changed the localization of GABARAP from the cytosol to the nucleus. The BDV P protein interaction with GABARAP delays the GABARAP‐mediated trafficking of GABA_A_ receptor to the cell membrane, which causes a block of GABA‐induced currents. It was also demonstrated that the last 27 amino acids of the C‐terminal part were essential for its binding to GABARAP [56].

Our iLIR‐ and hfAIM‐based analyses found a DEWDII motif at the C‐terminal part of the P protein between 195 and 200 amino acids. Based on our data, we suggest a GIR motif‐dependent interaction between P protein and GABARAP. We can also conclude that potentially the last 6 amino acids within the above‐identified 27 amino acid region might be responsible for the interaction.

As mentioned earlier, GST‐pull‐down experiments demonstrated the binding of Vif of HIV‐1 to all Atg8 family members, including GABARAP, GABARAP‐L1, and GABARAP‐L2 *in vitro*. While the authors did not study the importance of these interactions [44], interesting findings were published with the Nef (Negative factor) protein of HIV‐1. Nef controls the expression level of cell surface receptors by interfering with proteins in the endocytic and late secretory pathways [57]. Co‐IP and pull‐down assays verified that Nef binds to all GABARAP family members, but surprisingly not to the LC3 family members. NMR measurements testified that Nef binds to GABARAP through its S53 and F62 amino acids. Accumulation of Nef together with all GABARAP family members in cytoplasmic vesicular structures and at the plasma membrane was detected via live imaging. Plasma membrane accumulation was significantly disturbed with siRNA‐mediated silencing of GABARAP, GABARAPL1, and GABARAPL2. GABARAPs were found to be direct interaction partners of Nef, and this interaction is responsible for Nef plasma membrane localization and thus the Nef‐mediated pathogenesis of HIV‐1 [58]. Drug targeting of this interaction may disrupt Nef‐mediated pathogenesis and might provide clinical benefits.

## A case study: prediction and investigation of LIR motifs of SARS‐CoV‐2 proteins

In the ongoing pandemic, the question naturally arises: What do we know about the LIR motifs that may mediate SARS‐CoV‐2 viral proteins‐mAtg8 interactions?

The role of autophagy in SARS‐CoV‐2 coronavirus replication and intracellular trafficking is incompletely understood. We thus decided to investigate the LIR motifs of SARS‐CoV‐2 proteins and their role in the interaction with mAtg8 (primarily LC3B) proteins. In the next part, we want to demonstrate the efficacy of LIR prediction on three selected examples.

At the start of work, we analyzed the 29 SARS‐CoV‐2 proteins with two LIR prediction software programs. The iLIR program [59] identifies putative LIR motifs based on position‐specific scoring matrix (PSSM) values, which quantifies the reliability of prediction. The program also indicates if the motif falls into an intrinsically disordered protein region where the incidence of functional LIR motifs is high. The other software program, hfAIM [60], is suitable for genome‐wide analysis of LIR motifs and is characterized by stricter prediction conditions than the previous one, so the number of hits is lower. The parallel application of the two predictions allowed us to select only the most promising results for further analysis.

### Investigation of two nonstructural proteins of SARS‐CoV‐2, NSP15 and NSP12, identifies putative LIR motifs and colocalization with LC3B

NSP15 is a protein conserved in all coronaviruses with Mn^2+^‐dependent endoribonuclease activity. Its function is similar to that of *Xenopus laevis* endoU enzyme, which is a polyuridylate‐specific endoribonuclease involved in the processing of intron‐encoded small nucleolar RNAs. NSP15 endoribonuclease activity degrades polyuridine extension on 5′‐polyU‐containing negative‐sense RNAs, which would otherwise activate the host cell type I interferon response, and acts as a pathogen‐associated molecular pattern [61].

**Fig. 1:**
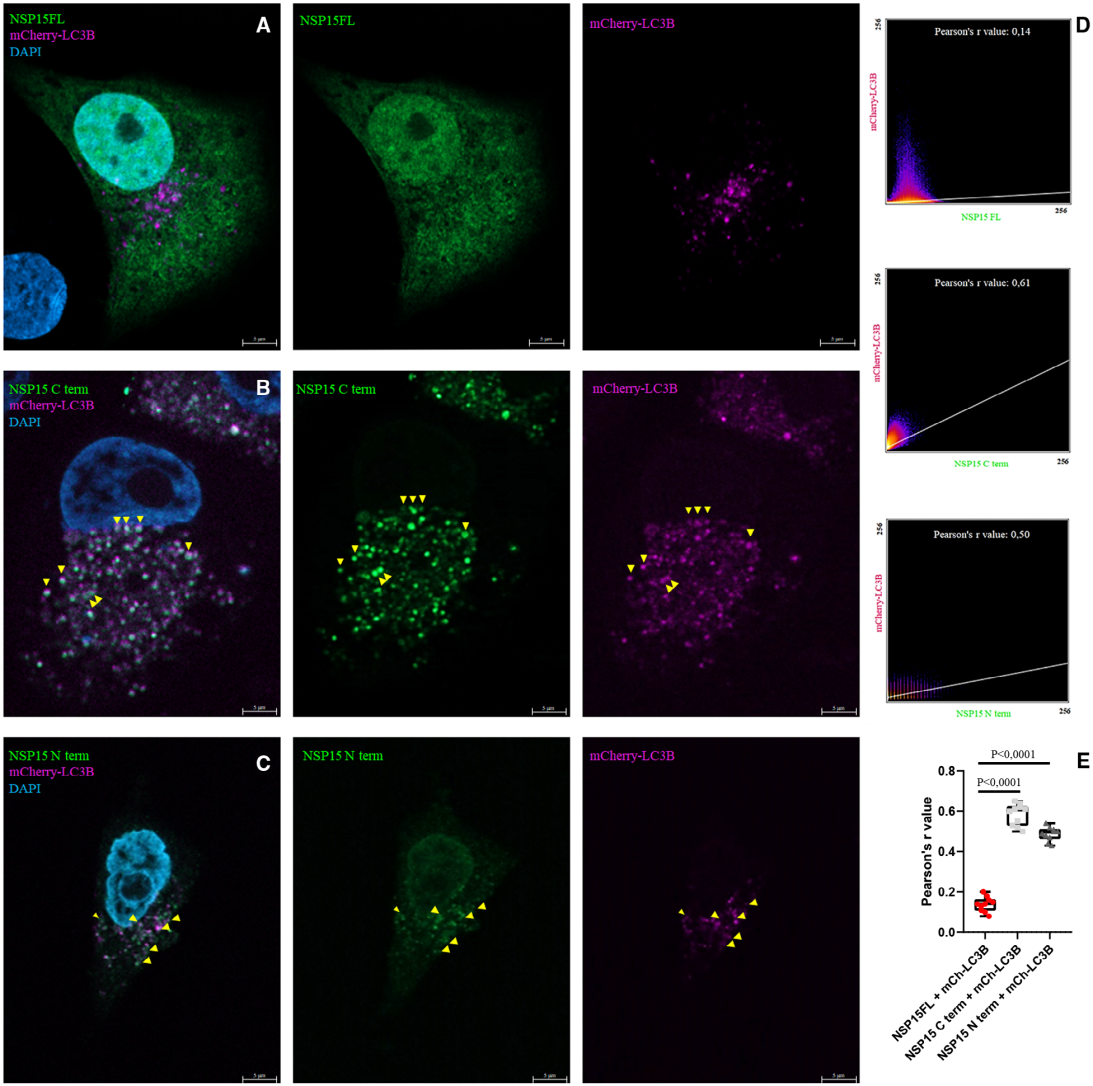
SARS‐CoV‐2 protein NSP15 N‐terminal and C‐terminal fragments colocalize with mCherry‐LC3B. MCF‐7 cells were cotransfected with GFP‐NSP15FL (A), GFP‐NSP15 C‐terminal part (B, amino acids: 273–347), GFP‐ NSP15 N‐terminal part (C, amino acids 1–224), and mCherry‐LC3B. The images display cell nuclei in blue (DAPI), NSP15 in green (GFP), and mCherry‐LC3B in magenta (mCherry). In panels (B) and (C), the yellow arrowheads indicate colocalization. In panel (D), scatter plots and Pearson's *r* values indicate colocalization in case of NSP15 C term and NSP15 N term. Representative images from at least two parallel experiments are shown. Panel (E) shows quantification of data, one‐way ANOVA test, *n* = 10. Scale bar, 5 μm.

Analysis of the NSP15 protein [SARS‐CoV‐2 NSP15 (Wuhan‐1) NCBI Reference Sequence: YP_009725310.1] with both iLIR and hfAIM software detected two putative LIR motifs (Table 2, N‐terminal LIR: aa. 210/211–216, C‐terminal LIR: aa. 299/300–305). In a previous study, Cao and Zhang performed a comparative analysis of the intracellular localization of GFP‐tagged NSP15 proteins of MHV, SARS‐CoV, and TGEV coronaviruses. SARS‐CoV and TGEV proteins were found to have a diffuse cytosolic distribution, while MHV NSP15 also showed adjacent perinuclear accumulation [62]. The N‐terminally tagged SARS‐CoV‐2 NSP15 protein showed diffuse cytoplasmic localization in MCF‐7 cells, like SARS‐CoV and TGEV NSP15 (Fig. 1A,D,E). Three regions were identified in the coronavirus NSP15 proteins by Cao and Zhang, which were responsible for the speckle‐like intracellular localization of each protein fragment. In our own studies, the identified LIR motifs may play a role in such localization, as both the N‐terminal part of the NSP15 protein containing the N‐terminal LIR motif and the C terminus showed colocalization with mCherry‐LC3B, and EGFP‐labeled NSP15 fragments marked autophagosome‐like vacuoles (Fig. 1B–E). It is important to note that the C‐terminal LIR motif of SARS‐CoV‐2 coincides exactly with the short protein region identified by Cao and Zhang as responsible for speckle formation.

**Table 2:** LIR motif prediction in SARS‐CoV‐2 NSP15, NSP12, and ORF9B proteins with hfAIM and iLIR software, including an ANCHOR prediction of whether the putative LIR motif is present in an unstructured region of the given protein.

hfAIM prediction			iLIR prediction				
Protein name	Predicted motif	Hit	Protein name	Predicted motif	Hit	PSSM score	LIR in Anchor
NSP15	X[DE][DE][WFY][ADCQEIGNLMFPSTWYV]X[LIV]	(299)LDDFVEI(305)	NSP15	xLIR	(300)DDFVEI(305)	19	No
	[DE]X[DE][WFY][ADCQEIGNLMFPSTWYV]X[LIV]	(210)EIDFLEL(216)		WxxL	(211)IDFLEL(216)	13	No
				WxxL	(322)IDYTEI(327)	14	No
NSP12	X[DE][DE][WFY][ADCQEIGNLMFPSTWYV]X[LIV]	(823)GDDYVYL(829)	NSP12	WxxL	(824)DDYVYL(829)	18	No
				WxxL	(266)IKWDLL(271)	14	No
ORF9B	X[DE][DE][WFY][ADCQEIGNLMFPSTWYV]X[LIV]	(88)PDEFVVV(94)	ORF9B	xLIR	(89)DEFVVV(94)	15	No

**Fig. 2:**
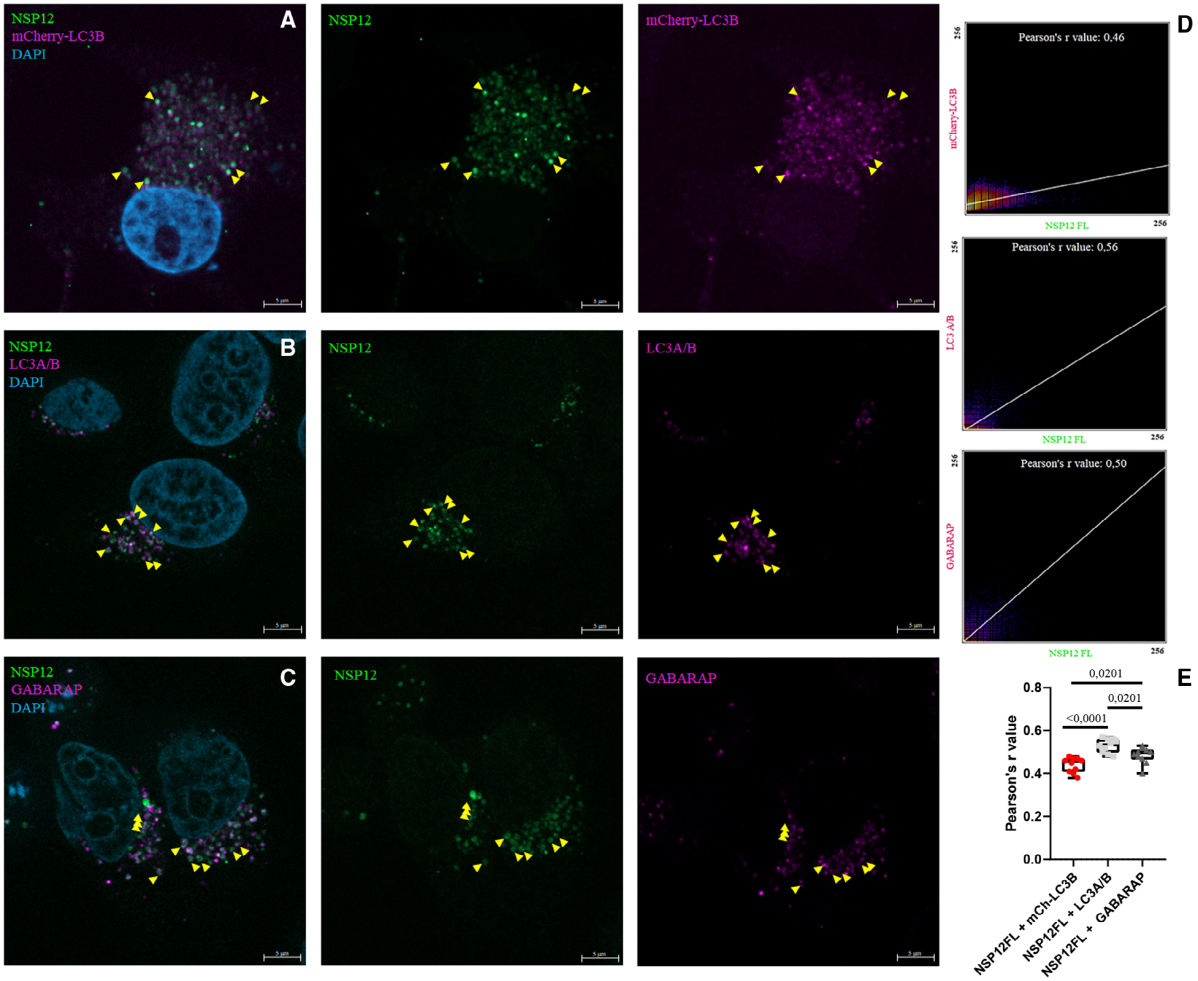
SARS‐CoV‐2 protein NSP12 colocalizes with mCherry‐LC3B and endogenous LC3A/B and GABARAP. MCF‐7 cells were cotransfected with GFP‐NSP12FL and mCherry‐LC3B. Anti‐LC3A/B and anti‐GABARAP antibodies were used at 1 : 250 dilution. The images display cell nuclei in blue (DAPI), NSP12 in green (GFP), and mCherry‐LC3B, LC3A/B and GABARAP in magenta. In panels (A), (B), and (C), yellow arrowheads indicate colocalization. In panel (D), scatter plots and Pearson's *r* values indicate colocalization. Representative images from at least two parallel experiments are shown. Panel (E) shows quantification of data, one‐way ANOVA test, *n* = 10. Scale bar, 5 μm.

The coronavirus RNA‐dependent RNA polymerase (RdRp) is the nonstructural NSP12 protein, which as a member of the holo‐RdRp complex (NSP7/NSP8/NSP12) is responsible for the synthesis of viral RNAs [63]. SARS‐CoV‐2 NSP12 (NCBI Reference Sequence: YP_009725307.1) is the target enzyme for antiviral agents such as remdesivir [64]. In our prediction, a LIR motif was detected in the NSP12 protein by both iLIR and hfAIM software (Table 2, NSP12 LIR: 823/824–829). This motif is at the border of the ‘PALM’ and ‘THUMB’ regions of NSP12, and this region may be accessible by mAtg8 according to the recently published cryo‐EM structure of the holoenzyme [65]. Our experiments showed that N‐terminally EGFP‐labeled NSP12 indeed colocalizes with the mCherry‐LC3B autophagosome marker (Fig. 2A,D,E). We also examined the colocalization of EGFP‐NSP12 with endogenous LC3A/B and GABARAP proteins and saw a similar colocalization as in the case of mCherry‐LC3B (Fig. 2B–E).

### SARS‐CoV‐2 ORF9b protein contains a putative LIR but it does not colocalize with LC3B

Mitochondrial and mitochondrial‐associated membrane (MAM) on ER are targets for binding of the coronavirus SARS‐CoV ORF9b protein [66]. This has been shown to play a role in the degradation of mitochondrial antiviral signaling protein (MAVS), directing its ubiquitination and degradation. It is also possible that by binding to the ER, the protein forms a bridge between the mitochondria and the ER and then initiates mitophagy. By degrading MAVS/TRAF‐3/TRAF‐6 and inducing mitophagy, ORF9b significantly reduces cellular antiviral defense responses.

A similar activity can be attributed to the SARS‐CoV‐2 ORF9b protein. According to a recent result, this protein binds to the TOM70 adapter molecule localized in the mitochondrial outer membrane, inhibiting its function and thus the type I interferon response (IFN‐I) [67].

**Fig. 3:**
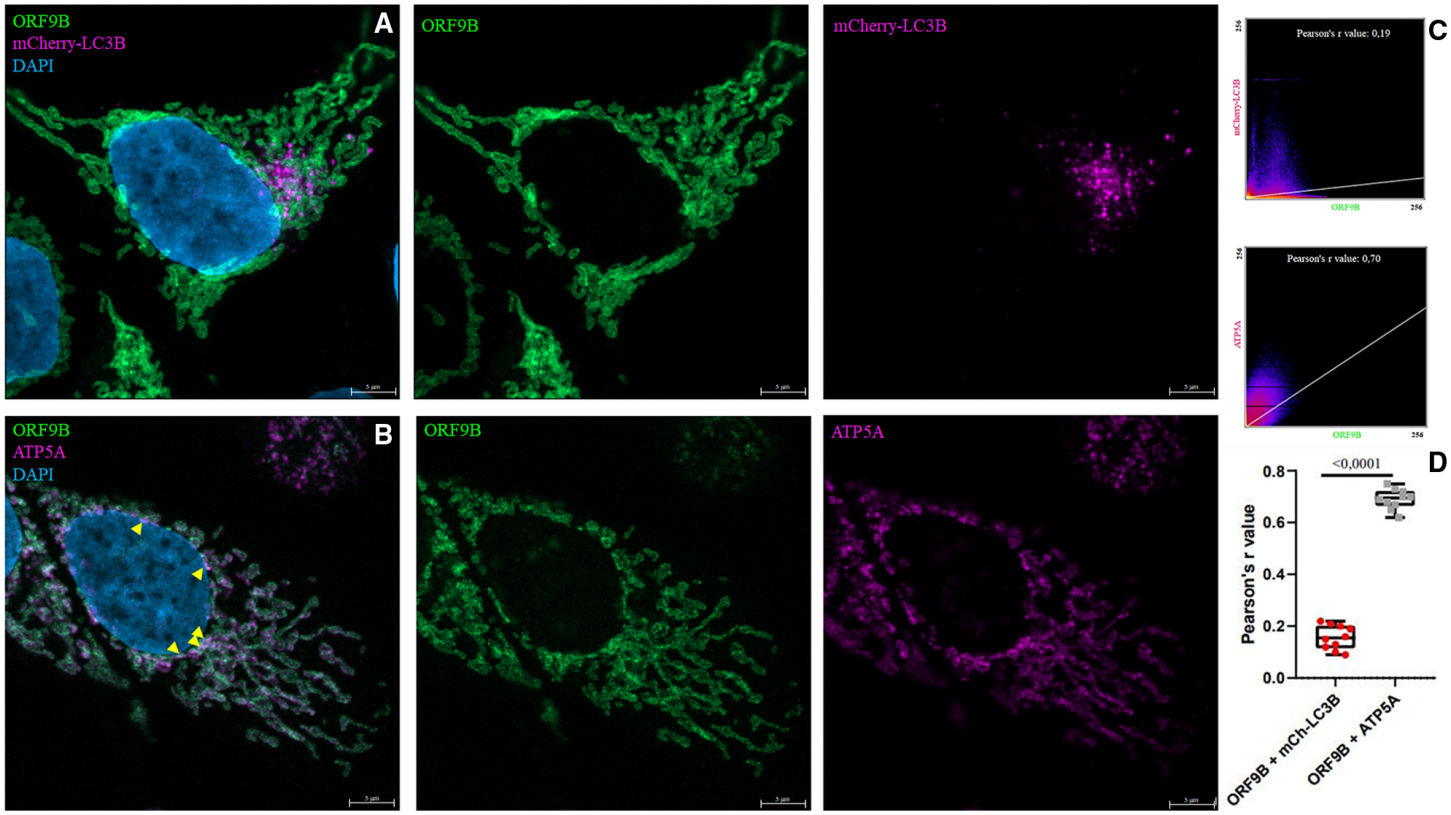
SARS‐CoV‐2 protein ORF9B does not colocalize with mCherry‐LC3B. MCF‐7 cells were cotransfected with GFP‐ORF9B and mCherry‐LC3B. Anti‐ATP5A antibody was used at 1 500 dilution. The images display cell nuclei in blue (DAPI), ORF9B in green (GFP), and mCherry‐LC3B (A) and ATP5A (B) in magenta (mCherry or Cy5), respectively. In panel (B), white signals and yellow arrowheads indicate colocalization. In panel (C), scatter plots and Pearson's *r* values indicate colocalization in case of ATP5A. Representative images from at least two parallel experiments are shown. Panel (D) shows quantification of data, two‐tailed unpaired *t*‐test, *n* = 10. Scale bar, 5 μm.

LIR prediction for the SARS‐CoV‐2 ORF9b (UniProtKB – P0DTD2 (ORF9B_SARS2)) protein also gave a promising result: Both software programs predicted a LIR motif in the flexible C terminus of the molecule (Table 2, aa. 88/89–94). The intracellular localization of EGFP‐ORF9b was the same as in previously published results: ER and mitochondrial localization were detectable. Somewhat surprisingly, unlike in the case of NSP12 and NSP15, no colocalization was detected with the mCherry‐LC3B marker (Fig. 3A,C,D), while colocalization with mitochondria was verified by colocalization with ATP5 protein located in the mitochondrial inner membrane (Fig. 3B–D). Thus, the predicted LIR may not be functional or perhaps it becomes exposed only under special circumstances, warranting further experiments.

According to a previous publication, the SARS‐CoV protein ORF9b induced autophagosome formation in an Atg5‐dependent manner [66]. Interestingly, this was not reflected at all in the number and size of mCherry‐LC3B‐labeled autophagosomes in our experiment. However, Shi et al. also noted that autophagy induction was not associated with an increase in LC3B‐II levels in their case either, suggesting that other mAtg8 protein(s) might play a role in this process.

## Conclusions

In this review, we summarized well‐characterized interactions of bacterial, parasitic, and viral proteins with Atg8 family proteins, as well as the outcome of these interactions on autophagy and/or pathogen replication. We also discussed the value of prediction software and the research methodology in the study of pathogen protein‐Atg8 family protein interactions, with reference to recently published results and with selected examples of potential LIR motif‐containing SARS‐CoV‐2 proteins. We also demonstrated the utility of hfAIM and iLIR databases regarding LIR motif prediction on SARS‐CoV‐2 proteins. These selected examples of *in silico* methods to predict LIR motifs in viral proteins can be tested experimentally (e.g., colocalization with mAtg8 by microscopy with colocalization analysis, and biochemical tests for interaction including co‐IP and pull‐down assays). Importantly, further verification using LIR mutant proteins is critical. The establishment of LIR mutant pathogens is also practical because it supports improved analysis of mechanisms. The identification of new LIR motif‐dependent‐mAtg8 interactions may have significant therapeutic value: It is clearly a druggable target, which, as we have already seen for Plasmodium, may lead to the development of new antipathogenic agents.

## Materials and methods

### Cell culture

MCF‐7 cells were cultured in Dulbecco's modified Eagle's medium (Sigma, Cat#D6429, Budapest, Hungary) supplemented with 10% FBS (Gibco HI FBS, Dublin, Ireland) and MycoZap™ Plus‐CL (500× concentrated solution) at a temperature of 37 °C and 5% CO_2_ level.

### Transfection

MCF‐7 cells were transiently transfected with 3 µg DNA, using 3 µL Lipofectamine 2000 transfection reagent (Invitrogen™, Cat#11668019, Waltham, MA, USA) in 2 mL Opti‐MEM according to the manufacturer's instructions.

### Microscopy preparation of transfected cells

Cells were grown on cover glasses and fixed with 4% paraformaldehyde (PFA) in PBS buffer at room temperature for 10 min, washed with 1× PBS and stained with 100× DAPI for 20 min.

### Immunofluorescence

Cells were grown on cover glasses and fixed with 4% paraformaldehyde (PFA) in PBS buffer at room temperature for 10 min, permeabilized with −20 °C methanol for 10 min, and then blocked with blocking buffer (1% BSA + 0.1% PBST) for 1 h at room temperature. Incubation with primary and secondary antibodies was performed in blocking buffer for 1 h at room temperature. The primary antibodies used were anti‐LC3A/B rabbit monoclonal antibody (Cell Signaling Technology, Cat #1274, 1 : 250, Danvers, MA, USA) and anti‐GABARAP + GABARAPL1 + GABARAPL2 rabbit monoclonal antibody (Abcam, Cat# ab109364, 1 : 250, Cambridge, UK). The secondary antibody used was Goat anti‐Rabbit IgG (H + L) Cross‐Adsorbed Secondary Antibody, Alexa Fluor 660 (Thermo Fisher, A‐21074, 1 : 500, Waltham, MA, USA).

### Confocal microscopy

Confocal images were captured on a Zeiss LSM880 microscope (Jena, Germany), equipped with a 63×, 1.4‐NA oil‐immersion lens. Images were processed in ZEN Lite 3.1, Photoshop CS3 (Adobe, San Jose, CA, USA) and imagej (NIH, Bethesda, MD, USA). Colocalization was measured with imagej software (National Institutes of Health) by calculating the Pearson correlation coefficient (Pearson's *r* value) using the Coloc2 plugin. Pearson's *r* value data from at least two independent experiments were imported to graphpad prism 8.0.1, graphpad software, San Diego, CA, USA and we confirmed normality of data distribution. After this, we calculated *P* values with the appropriate statistical tests: one‐way ANOVA or unpaired two‐tailed *t*‐test. *P* values are indicated over the clasps linking data pairs in each diagram.

## Conflict of interest

The authors declare no conflict of interest.

## Author contributions

DT drafted and wrote the manuscript and performed experiments. GVH wrote and revised the manuscript; GJ obtained funding, oversaw research, and revised the manuscript. All authors have read and agreed to the published version of the manuscript.

## Data Availability

All original data are accessible upon request.
